# A manually annotated *Actinidia chinensis* var. *chinensis* (kiwifruit) genome highlights the challenges associated with draft genomes and gene prediction in plants

**DOI:** 10.1186/s12864-018-4656-3

**Published:** 2018-04-16

**Authors:** Sarah M. Pilkington, Ross Crowhurst, Elena Hilario, Simona Nardozza, Lena Fraser, Yongyan Peng, Kularajathevan Gunaseelan, Robert Simpson, Jibran Tahir, Simon C. Deroles, Kerry Templeton, Zhiwei Luo, Marcus Davy, Canhong Cheng, Mark McNeilage, Davide Scaglione, Yifei Liu, Qiong Zhang, Paul Datson, Nihal De Silva, Susan E. Gardiner, Heather Bassett, David Chagné, John McCallum, Helge Dzierzon, Cecilia Deng, Yen-Yi Wang, Lorna Barron, Kelvina Manako, Judith Bowen, Toshi M. Foster, Zoe A. Erridge, Heather Tiffin, Chethi N. Waite, Kevin M. Davies, Ella P. Grierson, William A. Laing, Rebecca Kirk, Xiuyin Chen, Marion Wood, Mirco Montefiori, David A. Brummell, Kathy E. Schwinn, Andrew Catanach, Christina Fullerton, Dawei Li, Sathiyamoorthy Meiyalaghan, Niels Nieuwenhuizen, Nicola Read, Roneel Prakash, Don Hunter, Huaibi Zhang, Marian McKenzie, Mareike Knäbel, Alastair Harris, Andrew C. Allan, Andrew Gleave, Angela Chen, Bart J. Janssen, Blue Plunkett, Charles Ampomah-Dwamena, Charlotte Voogd, Davin Leif, Declan Lafferty, Edwige J. F. Souleyre, Erika Varkonyi-Gasic, Francesco Gambi, Jenny Hanley, Jia-Long Yao, Joey Cheung, Karine M. David, Ben Warren, Ken Marsh, Kimberley C. Snowden, Kui Lin-Wang, Lara Brian, Marcela Martinez-Sanchez, Mindy Wang, Nadeesha Ileperuma, Nikolai Macnee, Robert Campin, Peter McAtee, Revel S. M. Drummond, Richard V. Espley, Hilary S. Ireland, Rongmei Wu, Ross G. Atkinson, Sakuntala Karunairetnam, Sean Bulley, Shayhan Chunkath, Zac Hanley, Roy Storey, Amali H. Thrimawithana, Susan Thomson, Charles David, Raffaele Testolin, Hongwen Huang, Roger P. Hellens, Robert J. Schaffer

**Affiliations:** 1 The New Zealand Institute for Plant & Food Research Ltd (PFR), Private Bag 92169, Auckland, 1142 New Zealand; 2 School of Biological Sciences, University of Auckland, Private Bag 92019, Auckland, 1142 New Zealand; 3 PFR, Private Bag 11600, Palmerston North, 4442 New Zealand; 4 PFR, 412 No 1 Road, Te Puke, Bay of Plenty 3182 New Zealand; 5 IGA Technology Services, Parco Scientifico e Tecnologico, Udine, Italy; 6 South China Botanic Gardens, Chinese Academy of Sciences, Guangzhou, 510650 Guangdong China; 7 Key Laboratory of Plant Germplasm Enhancement and Specialty Agriculture, Botanical Garden, Chinese Academy of Sciences, Wuhan, 430074 Wuhan China; 8 PFR, Private Bag 4704, Christchurch, 8140 New Zealand; 9 Department of Agricultural and Environmental Sciences, University of Udine, Via delle Scienze 208, 33100 Udine, Italy; 10 Institute for Future Environments, Queensland University of Technology (QUT), Brisbane, 4001 Australia

**Keywords:** Manual annotation, Genome sequencing, *Actinidia chinensis*

## Abstract

**Background:**

Most published genome sequences are drafts, and most are dominated by computational gene prediction. Draft genomes typically incorporate considerable sequence data that are not assigned to chromosomes, and predicted genes without quality confidence measures. The current *Actinidia chinensis* (kiwifruit) ‘Hongyang’ draft genome has 164 Mb of sequences unassigned to pseudo-chromosomes, and omissions have been identified in the gene models.

**Results:**

A second genome of an *A. chinensis* (genotype Red5) was fully sequenced. This new sequence resulted in a 554.0 Mb assembly with all but 6 Mb assigned to pseudo-chromosomes. Pseudo-chromosomal comparisons showed a considerable number of translocation events have occurred following a whole genome duplication (WGD) event some consistent with centromeric Robertsonian-like translocations. RNA sequencing data from 12 tissues and ab initio analysis informed a genome-wide manual annotation, using the WebApollo tool. In total, 33,044 gene loci represented by 33,123 isoforms were identified, named and tagged for quality of evidential support. Of these 3114 (9.4%) were identical to a protein within ‘Hongyang’ The Kiwifruit Information Resource (KIR v2). Some proportion of the differences will be varietal polymorphisms. However, as most computationally predicted Red5 models required manual re-annotation this proportion is expected to be small. The quality of the new gene models was tested by fully sequencing 550 cloned ‘Hort16A’ cDNAs and comparing with the predicted protein models for Red5 and both the original ‘Hongyang’ assembly and the revised annotation from KIR v2. Only 48.9% and 63.5% of the cDNAs had a match with 90% identity or better to the original and revised ‘Hongyang’ annotation, respectively, compared with 90.9% to the Red5 models.

**Conclusions:**

Our study highlights the need to take a cautious approach to draft genomes and computationally predicted genes. Our use of the manual annotation tool WebApollo facilitated manual checking and correction of gene models enabling improvement of computational prediction. This utility was especially relevant for certain types of gene families such as the EXPANSIN like genes. Finally, this high quality gene set will supply the kiwifruit and general plant community with a new tool for genomics and other comparative analysis.

**Electronic supplementary material:**

The online version of this article (10.1186/s12864-018-4656-3) contains supplementary material, which is available to authorized users.

## Background

The time, effort and cost of obtaining whole genome sequences has reduced dramatically since the publishing of the first whole plant genome for *Arabidopsis thaliana* in 2000 [1]. As a result, more than 100 plant genomes have now been sequenced, including those for a number of fruit crops of worldwide horticultural importance, such as *Vitis vinifera* (grape) [2], *Carica papaya* (papaya) [3], *Malus* x *domestica* (apple) [4], *Fragaria vesca* (strawberry) [5], *Solanum lycopersicum* L. (tomato) [6], *Musa acuminata* (banana) [7], *Citrus sinensis* (orange) [8], and *Pyrus comunis* L. (European pear) [9]. However, there are still many challenges for plant genome assembly including fragmentation, large numbers of contigs, mis-assembly and the polyploid nature of many plant species, contribute to large amounts of sequence remaining unassigned to chromosomes in many genomes and thus impact the quality of the gene annotation within them [10, 11]. This is now being addressed with new improved versions of genomes appearing in the literature [12, 13].

The *A. chinensis* draft genome [14] represented a significant step forward for kiwifruit researchers. However, as is typical for draft whole genome sequences, a significant proportion of the scaffolds was unassigned to chromosomes, and mis-assemblies have been subsequently identified: Scaglione and colleagues were the first to identify scaffold misplacements and revealed significant discrepancies that indicated scaffold mis-assignments in chromosomes (Chr) 6, 10, 16, 18, 19, 20 and 21 [15]. The most significant discrepancy was 4.5 Mb of scaffolds attributed to Chr10 that mapped unambiguously to Chr16. Zhang and colleagues also reported the possibility of scaffold anchoring errors and suggested that the draft genome contained many inter-chromosomal misplacements [16].

The ‘Hongyang’ genome sequence was annotated using a combination of computer annotation, Expressed Sequence Tag (EST) sequence information from publicly available databases and in-house RNA sequencing (RNA-Seq), which resulted in 39,040 predicted genes [14]. The predicted ‘Hongyang’ gene models have recently been shown to omit key published *EXPANSIN* (*EXP*) genes [17]. These missing genes may be due to errors in the genome assembly itself, caused by introduced stop codons nullifying a prediction. Although, the majority (97% [14]) of the *EXP* ESTs are found in the ‘Hongyang’ genome sequence, most were not found in the annotated gene list. This indicates that the rules set for inclusion of a predicted gene in the published gene set may have been too conservative, and that re-annotation of the genome sequence is necessary to improve representation within the predicted gene set currently available. To that end, an extensive revision of the ‘Hongyang’ annotation was performed by Yue and colleagues [18]. Their efforts yielded a much improved annotation for ‘Hongyang’ as well as providing further information on splice variants, predicted metabolic pathways and protein-protein interactions. In other species, gene models constantly evolve, with *Arabidopsis* now on its 11th release [19]. Computational re-annotation [20] of the strawberry genome [5] increased the total number of gene model predictions by 2286 predictions.

Most commonly, genome annotation methods have been computationally derived [4, 5], although research communities are increasingly combining computer annotation methods with manual annotation that allows researchers to improve individual gene models within the genome. The software package WebApollo [21] has been used for gene annotation initiatives in a number of species, including *Caenorhabditis elegans* [22], yeast [23] and honey bee [21], as well as *Arabidopsis* [24]. Community annotation leverages the expert knowledge within a community to identify and correct errors in computational predictions and insert models missed by those computational approaches. At present, manual curation of genomes is rare, but could become increasingly common as researchers recognise that computational assembly and annotation alone are not sufficient.

In our study, three research teams pooled genetic mapping and sequence resources to generate a genome of a second *A. chinensis* genotype, Red5, with higher homozygosity than ‘Hongyang’. This information was combined with EST sequencing results [25] and RNA-Seq data and made available to annotators via WebApollo to facilitate manual annotation of the new genome. The whole genome was manually annotated, resulting in what we believe to be a considerable improvement in allocating previously unallocated regions and in gene model quality as compared to existing resources.

## Results

### Assembly of a second genome of *Actinidia chinensis var. chinensis*

**Fig. 1:**
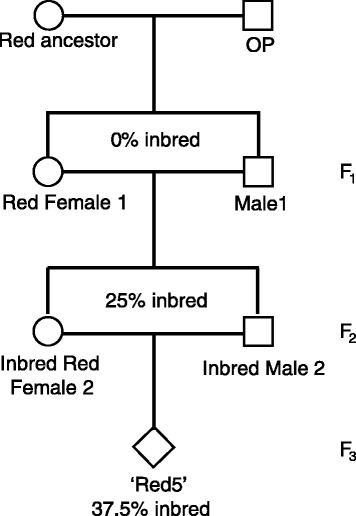
The pedigree tree for Red5 kiwifruit. Two siblings were crossed from open-pollinated seed from a red *Actinidia chinensis*. An F_2_ sibling cross from two individuals from this population resulted in Red5 with a predicted inbreeding coefficient of 37.5%

To generate a new *Actinidia chinensis* genome, a diploid F3 sibcross individual Red5, with a predicted inbreeding coefficient of 37.5%, was chosen for sequencing (Fig. 1). An anytag-based assembly of paired-end Illumina reads generated 46,117,212 fragments with an N50 of 275 bases [26]. Assembly of these fragments using a long insert library (Roche 454 GS-FLX – 4 kb) using Newbler produced 39,868 contigs. Subsequent stepwise scaffolding using SSPACE2 [27] with Illumina long-range insert libraries of 4, 9 and 13 kb yielded 39,825, 8688 and 3887 scaffolds, respectively. After two iterations of gap closure the final assembly consisted of 3887 scaffolds with a total length of 550.5 Mb. The N50 was 623.8 kb with L50 of 240 scaffolds, an N90 of 140.7 kb with L90 of 941 scaffolds, and 3.57% N content, with the longest scaffold being 4.43 Mb.

**Fig. 2:**
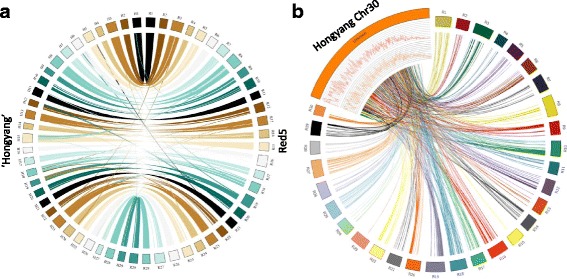
**a**. Genome construction with respect to the original submitted *Actinidia chinensis* ‘Hongyang’ genome, showing the rearrangement of some assigned chromosomes and **b**. the distribution of unallocated sequence (Chromosome 30) to the rest of the genome

The genetic linkage map of Scaglione and colleagues [15], which included markers from Fraser and colleagues [28], augmented by BLAST walking comparison between Red5 and scaffolds of ‘Hongyang’ enabled anchoring of 2727 scaffolds (Table 1) comprising 547.9 Mb, to 29 linkage groups. The remaining 1206 unanchored scaffolds containing 5.91 Mb with an N50 of 5.36 kb were concatenated to form a composite entity hereafter referred to as ‘Chr30’ for the purposes of subsequent manual annotation of the entire genome sequence. Note, upon submission to NCBI Genbank scaffolds assigned to ‘Chr30’ were submitted as individual (non-concatenated) scaffolds according to NCBI Genbank submission policy. Estimates of genome size based on K-mer analysis indicated a genome size of 705 Mb (preQC) or 742 Mb (jellyfish). These align with estimates from flow cytometry [29] that report the genome to be 758 Mb in size. The assembly therefore represents ~ 73% of the estimated genome size with 98.9% of assembled scaffolds (72% of the estimated genome size) assigned to chromosomes. This is a considerable improvement from the original ‘Hongyang’ draft genome, which had 164 Mb unassigned (Fig. 2).

**Table 1:** Construction metrics for the *Actinidia chinensis* genome

Chromosome	Size (Mb)	Number of Scaffolds	Scaffold N50 (Kb)	Longest Scaffold (Mb)	Manually Annotated Genes		
					Number of Genes	Number of Transcripts	Genes Per 100 Kb
1	18.6	94	595.4	1.51	1125	1133	6.06
2	14.6	83	536.6	1.41	1091	1094	7.46
3	21.7	92	1154.9	2.96	1726	1729	7.94
4	13.8	75	420.4	2.39	781	781	5.67
5	18.6	113	655.2	4.44	960	976	5.16
6	17.4	109	474.8	1.56	1059	1069	6.09
7	20.0	99	666.0	1.82	1068	1075	5.33
8	26.1	134	779.6	1.68	1448	1459	5.55
9	16.6	66	598.4	1.95	1118	1123	6.74
10	19.3	111	413.0	0.95	986	994	5.10
11	16.9	74	520.3	1.26	1119	1137	6.63
12	19.2	122	480.2	1.42	1053	1054	5.49
13	19.5	77	926.8	1.65	1388	1402	7.11
14	17.9	75	651.2	2.32	1106	1129	6.18
15	15.9	67	707.9	2.13	1106	1121	6.94
16	23.8	152	388.5	1.46	1252	1255	5.26
17	17.4	100	387.2	0.91	931	934	5.34
18	20.7	85	850.4	2.80	1213	1219	5.85
19	15.4	117	345.9	2.12	653	656	4.24
20	17.9	89	512.5	1.53	1055	1056	5.88
21	17.3	71	871.2	2.16	1046	1064	6.04
22	18.9	105	462.5	1.42	1092	1096	5.76
23	27.7	79	802.6	2.15	2324	2327	8.39
24	17.8	59	1032.0	3.02	1198	1201	6.72
25	19.6	83	966.1	2.45	1008	1010	5.14
26	20.4	79	1086.4	3.41	1237	1247	6.07
27	21.0	112	461.8	1.27	1013	1040	4.83
28	15.8	75	964.5	2.80	1011	1012	6.38
29	18.0	130	402.3	0.69	993	1002	5.53
Total for Chrs 1–29	548.0	2727	575.6	4.44	33,160	33,395	6.05
*Unassigned* 30	5.9	1206	5.4	0.66	97	97	1.64
Total	553.9	3933			33,257	33,492	6.00

### Evaluation of genome assembly

To evaluate genome assembly accuracy we assessed mapping back of paired end reads to the assembly and compared the assembled contigs for 22 clones from a BAC library of *A. chinensis* Red Female 1 (Fig. 1). These contigs resulted from sequencing using a different technology (454) and different assembly path (Newbler). Rates of discordant alignment of input paired end reads mapping once to the whole genome sequence was low (0.33 to 2.24%) (Additional file 1). Alignment of these BAC clone contig assemblies to chromosome 25 supported the assembly of Red5. The alignments (Additional file 2) show a close correspondence between the BAC clone assemblies and the whole genome assembly of Red5 in this region of chromosome 25. Additionally, the alignment of read pairs from the 9Kb LIMP library was assessed and visualised using hagfish_blockplot from the ‘hagfish’ software (https://github.com/mfiers/hagfish/). The majority of alignments displayed green indicating the read pairs from the 9Kb LIMP library aligned to the chromosome sequences with the default bounds determined by ‘hagfish’ (Additional file 3). As expected for assembly from short read data there were also regions depicted in pinkish-red suggesting that the mate pairs aligned to the genome in these regions outside the expected distances. Such regions will occur for example when repeats are compressed into a consensus leading to a compression in the whole genome assembly sequence relative to the physical genome sequences.

To evaluate genome completeness BUSCO analysis [30] was undertaken. For purposes of comparison, these same analyses were repeated using the published chromosomal sequences for ‘Hongyang’ [14]. Red5 contained 1364 (94.7%) ‘complete’ BUSCOs, of which 1022 (75.0%) were reported as ‘complete and single-copy’, while 342 (25%) were reported as ‘complete and duplicated’ with 27 reported as fragmented and 49 reported as missing. In comparison, ‘Hongyang’ contained 1358 (94.3%) ‘complete’ BUSCOs, with 22 (1.5%) reported as fragmented and 60 (4.2%) reported as missing. Of the BUSCOs reported as ‘complete’ in ‘Hongyang’, 987 (68.5%) were reported as ‘complete and single copy’, while 371 were reported as ‘complete and duplicated’. When the 47,384 *A. chinensis* EST sequences in NCBI GenBank were mapped to the Red5 chromosomes, only 580 had no homology, with 2368 ESTs aligned at less than 74% match. In comparison, when the ESTs were aligned to the published chromosomes of ‘Hongyang’, 3295 had no homology to any region, suggesting Red5 and Hongyang have a comparable gene space assembly. When RNA-Seq data from a range of tissues (Table 2) were mapped to the chromosome assembly of Red5, an average of 91.95% of 316.2 million RNA-Seq reads mapped uniquely (ranging from 88.19% to 94.56% for the different tissues). 6.42% total reads mapped to multiple locations.

**Table 2:** Tissue for RNA-Seq data for the *Actinidia chinensis* genome

Red5 tissue description	Number of reads
tissue culture whole plant	16 M
orchard plant growing bud	16 M
orchard plant flower	19 M
orchard fruit 0 DAFB^1^	18 M
orchard fruit 14 DAFB^1^	0.2 M
orchard fruit 60 DAFB^1^	14 M
orchard fruit 76 DAFB^1^	59 M
orchard fruit 139 DAFB^1^	60 M
glasshouse pot plant root tip	56 M
glasshouse pot plant root main	58 M

^1^*DAFB* days after full bloom

**Fig. 3:**
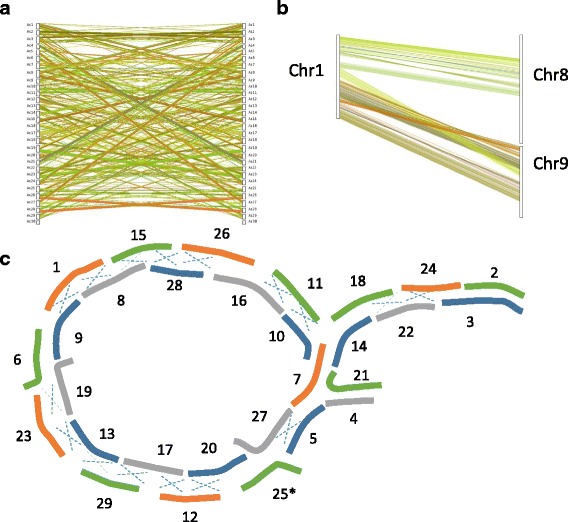
Duplicated regions in *Actinidia chinensis* Red5. **a**. whole genome lined up with whole genome at 85% homology. **b**. Alignments of Chr9 and Ch8 with Chr1, suggesting a translocation event has occurred. **c**. A schematic of the translocated chromosomes. A dashed “x” suggests a Robertsonian translocation has occurred at the centromeric regions. Chromosomes not aligned with a dashed “x” show regions of homology i.e. other proposed chromosomal rearrangements. Asterisk marks the sex chromosome (Chr25)

Global analysis comparing the new genome with itself revealed areas of similarity among different regions of the chromosomes (Fig. 3a). When these duplicated chromosomes were examined more closely it was found that many appeared to have Robertsonian–like centromeric translocations. This could be clearly seen, for example, in the duplicated Chr1, which showed homology to half of Chr8 and half of Chr9 (Fig. 3b). The paired chromosomes were arranged sequentially (Fig. 3c) and all but two had at least one translocation event. The only non-translocated chromosomes were Chr4 (homeologous to Chr21) and Chr2 (homeologous to Chr3) (Fig. 3c).

#### Manual curation of the predicted gene models

To develop gene models for the new genome, a WebApollo tool [21] was populated with the new genome sequence and the following tracks of evidence were added:

1. A scaffold assembly quality track which identified single N insertions, indicative of construction anomalies, and N repeats that marked the edges of scaffold boundaries
2. A repeat masker track that identified repetitive elements, including transposons
3. Computational gene prediction tracks consisting of the original ‘Hongyang’ gene models, and an ab initio gene prediction scan of the Red5 genome
4. ESTs from published EST sequencing libraries, and 12 tracks of RNA-Seq from diverse tissues from Red5 (Table 2).

An international consortium of annotators synthesised this information to produce a new gene model for each gene along the genome.

**Fig. 4:**
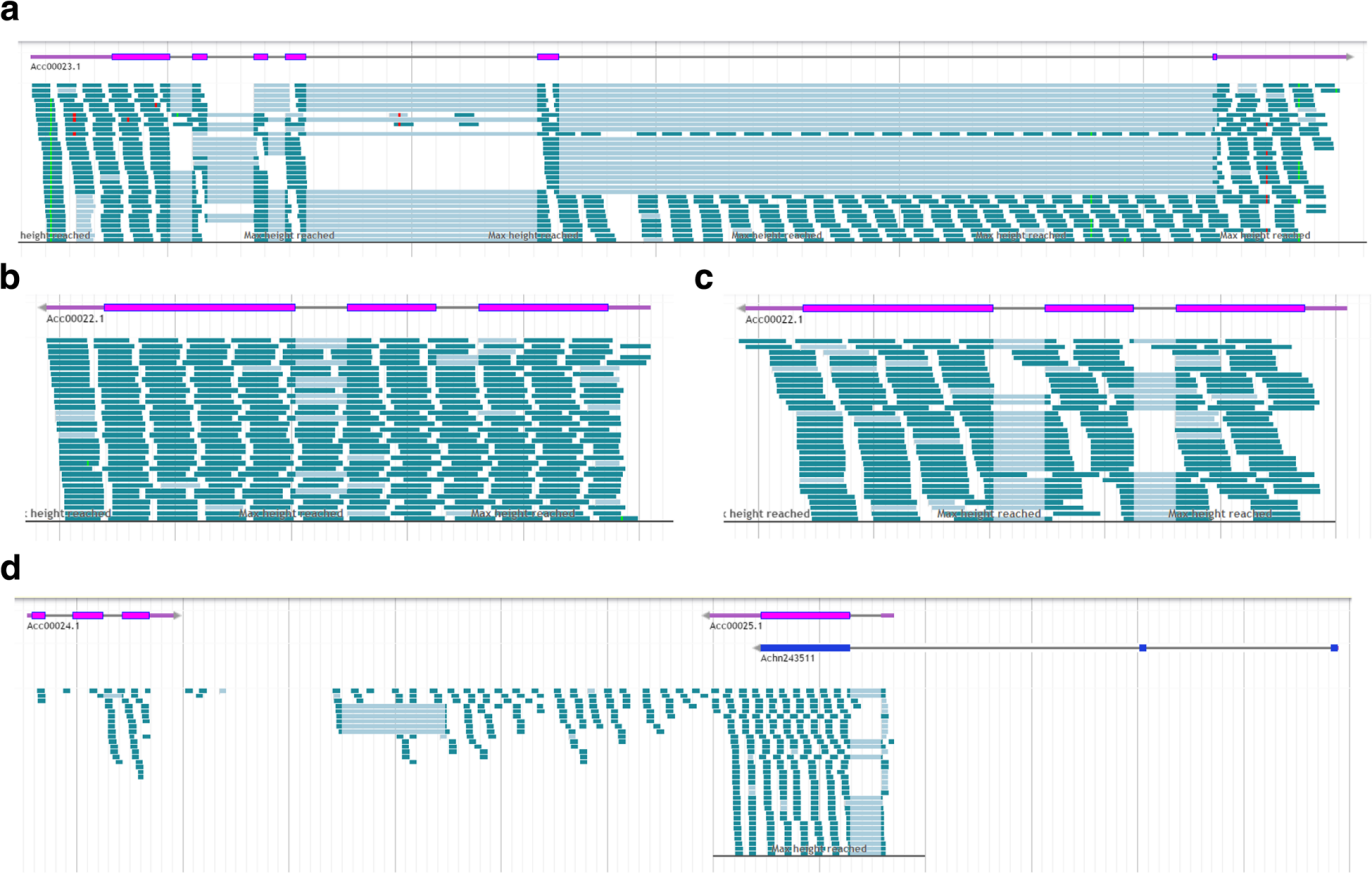
Examples of genes with transcription, and no transcription in intronic regions highlighting some of the challenges associated with manual annotation. **a** RNA-Seq reads in a single intron of gene Acc00023. **b** and **c** a comparison of RNA-Seq reads in two independent RNA-Seq libraries of gene Acc00022. B) RNA-Seq from a root library showing transcription across the intron and C) RNA-Seq from a flower library showing no intronic RNA-Seq reads. **d** Transcription between gene models Acc00024 and Acc00025, which could not be constructed into a gene model; the *Actinidia chinensis* ‘Hongyang’ predicted model line-up Achn243511 is shown in blue

During the annotation process, some models were relatively simple to predict while others were more difficult. Easy-to-annotate models had strong RNA-Seq support, clear intron-exon structures and a gradation in the number of RNA-Seq reads at the beginning and the end of the gene. These genes also often had an accurate computationally predicted gene model and had a good BLASTP match from GenBank that covered the majority of the gene. Conversely, there were many regions of the genome that were very difficult to annotate. These harder-to-annotate regions had conflicting, patchy, or even no RNA-Seq evidence, combined with conflicting or absent computational gene models (Fig. 4). The complexity of these gene regions was often confounded by genome structure caused by repetitive elements and/or anomalies in the genome sequence construction, as observed by a single “N” or scaffold boundaries in the quality track. Within the genome, the whole spectrum of combinations of these challenges were identified with occasional loss of open reading frame caused by the anomalies. To address this variation in confidence for each model, a quality tag was added to each of the gene models. A strongly supported high quality gene model was given a Q2 tag, while a model that was suspected to be incomplete or contain other errors was given a Q1 tag. Further tags based on whether the BLASTP alignment suggested it was full length (F) or a partial (P) gene were also added. A Q0 tag was given to computationally predicted genes that exhibited peptide homology to a GenBank protein, but no RNA-Seq evidence. Following the first pass of manual annotation, it was found that there was a high degree of variability among the 93 annotators in their interpretation of the evidence to create the models, especially for loci where evidence was ambiguous, sparse, or apparently contradictory. To address this variation, a second pass of the genome was undertaken by a smaller group of ‘expert’ annotators to standardise the annotation.

The manual annotation identified 33,044 gene loci, with 33,123 protein isoforms. The mean coding length of annotated genes was 1278 bases, while the mean locus length was 6071 bases (range 282–92,048 bases). Of the 33,123 loci, 32,967 (99.5%) were manually annotated with a single isoform, while 76 were annotated with two isoforms. One locus was annotated with four isoforms respectively. It should be noted that for loci with potentially more than one isoform individual isoforms were only annotated where they were cleanly defined from the RNA-Seq evidence. Otherwise a single model was submitted by annotators. Therefore it is likely that there will be further isoforms defined as further evidence is obtained. Of the 33,123 loci, 2485 were annotated by the community annotators as partials, with 1057 annotated as having 5′ truncations, and 817 as having 3′ truncations, while 611 loci were annotated as being partial without an indication of truncation direction. The total number of coding sequence regions (CDS) in annotated genes was 181,135 and contained 42.3 Mb of genome sequence (5.58% of the estimated genome size). There were 6514 loci (19.7%) annotated as containing a single CDS. The mean length of CDS regions in single CDS loci was 1004 bases. In comparison, the mean CDS length for all loci was 233 bases. The mean intron length within the coding regions of multiple exon-containing loci was 886 bases. The minimum intron length was 22 bases. The corresponding quality scores were assessed and 83.16% of gene models had a Q2 score, 14.62% had a Q1 score and 2.18% had a Q0 score.

Since genomes are always evolving, it is likely that different genomic structures will be observed across *Actinidia* species. Additionally, new improved versions of the genome annotation will be developed that incorporate the last 6 Mb of unassigned fragments. To address this, we named our genes each with a unique name that will be enduring and independent of chromosome location. We have used the nomenclature AccXXXXX with splice variants appended as ‘.1’ and ‘.2’ etc., and then appended chromosome location as a descriptor which can subsequently be changed, without changing the unique name of the gene. To assist the kiwifruit research community we provide a conversion table for the best reciprocal matches between our gene set and that within the Kiwifruit Information Resource (KIR) [18] as Additional file 4 and will also make this data available via our Git Repository (https://github.com/PlantandFoodResearch/Red5_WGS_Manual_Annotation).

In addition to the location, the sequence length, sequence type (cds, cdna, peptide), and manual annotation quality score were appended along with an internal database identifier and functional description. The manual quality score also contained the F and P labels based on BLAST match, so a point to note, is a gene with a manual quality score of 2 score (good RNA-Seq support) but a BLAST alignment indicating truncation could be scored as 2P with the direction of truncation indicated by using a 5 or 3 suffix, for example a 5 prime truncation would be scored ‘2P5’.

### Comparison of the Red5 gene set with the ‘Hongyang’ gene annotation

The manually annotated gene set was compared with the 39,040 published ‘Hongyang’ gene models originally published [14] (hereafter termed original ‘Hongyang’ model set) as well as to the 39,761 revised gene annotations [18] (hereafter termed revised ‘Hongyang’ model set). As ‘Hongyang’ is a different cultivar, polymorphisms are expected. To get a more accurate comparison, predicted protein sequences were used. When Red5 was used as query against the original ‘Hongyang’ model set as a database it was found that only 1973 (~ 6%) of the protein sequences for the 33,123 isoforms were identical in sequence and length to a ‘Hongyang’ predicted protein model [14]. We also detected instances where a Red5 model was perfectly contained within a ‘Hongyang’ model suggesting either the Red5 model is truncated, the ‘Hongyang’ model is over predicted or there is a genotypic difference between the two genotypes. The reverse situation where a ‘Hongyang’ model was perfectly contained in a Red5 model was also encountered. 882 (2.67%) of Red5 proteins were perfectly contained within a longer sequence of an original ‘Hongyang’ model while 828 (2.51%) Red5 proteins perfectly contained the sequence of an original ‘Hongyang’ model. When the revised ‘Hongyang’ model set [18] was employed as the database 3114 (9.4%) Red5 protein sequences were found to be identical in sequence and length to a protein within the revised ‘Hongyang’ model set while 927 and 1007 Red5 proteins respectively either were perfectly encapsulated within a revised ‘Hongyang’ model or perfectly encapsulated a revised ‘Hongyang’ model. We repeated the analysis in the reverse direction. When using Red5 as the database, 42% of the original ‘Hongyang’ model proteins and 54.2% of the revised ‘Hongyang’ model proteins possessed a match with identity of 90% or greater, showing a considerable number of genes have been changed firstly in the revised annotation and secondly in the manual annotation process.

Comparison of the Red5 to original ‘Hongyang’ models identified 1958 original ‘Hongyang’ models had identical sequence and identical length to the corresponding Red5 model. A further 1261 original ‘Hongyang’ models possessed identical protein sequence to a Red5 model but were shorter in length than the Red5 model while for 576 original ‘Hongyang’ models the reverse was true. As expected 3114 of the revised models were found to have identical sequence and identical length to the corresponding Red5 model. A further 1685 revised ‘Hongyang’ models possessed identical protein sequence to a Red5 model but were shorter in length compared to the Red5 model while for 553 revised ‘Hongyang’ models the reverse was true. To examine the relationship with less than perfect matching best reciprocal BLASTP matches between Red5 and ‘Hongyang’ protein datasets identified for 19,179 proteins [14] and 21,479 proteins [18]. When the lengths of predicted proteins identified as best reciprocal BLASTp matches were compared, 5542 and 4700 proteins respectively within original and revised ‘Hongyang’ genes, respectively, possessed a longer protein sequence length than the Red5 model. By comparison 13,482 and 16,551 Red5 protein were longer than their best reciprocal BLASTp match counterparts from original and revised ‘Hongyang’ model sets respectively.

Within both the original and revised ‘Hongyang’ gene sets 148 and 113 models were completely missing from the Red whole genome sequence. The identifiers for these models are listed in Additional file 5. For 1195 original and 587 revised ‘Hongyang’ models lacking a best reciprocal BLASTp match we found the CDS for these models to be encapsulated in the UTR regions of Red5 models. For a further 379 original and 82 revised ‘Hongyang’ models lacking protein:protein matches to Red5 proteins we found their CDS to overlap the 3 UTR of a Red5 model while for 362 original and 77 revised ‘Hongyang’ models the CDS was found to overlap the 5 UTR of a Red5 model. A further 3534 and 2034 models from the original and revised ‘Hongyang’ sets respectively were completely present in the whole genome sequence of Red5 but possessed no protein match to a Red5 gene model and did not align to a UTR region of a Red5 model. To identify if these models were missing from our annotation set due to lack of support from RNA-Seq evidence we merged BAM files for RNA-Seq libraries previous aligned to the Red5 whole genome sequence for purposes of assisting manual annotation. The CDS for the ‘Hongyang’ gene set of Yue and colleagues [18] was aligned to the Red5 whole genome sequence using GMAP [31] (version 2017–06-20), the resultant GFF3 output converted to Simplified Annotation Format (SAF) and RNA-Seq read counts to these features extracted using featureCounts [32].

Of the 2034 revised ‘Hongyang’ models that perfectly aligned to the Red5 whole genome sequence but for which there was not protein match of any kind, 535 aligned to regions of the Red5 genome where there was no aligned RNA-Seq and thus would have been unlikely to be manually annotated as a result. To further examine the RNA-Seq alignment of the remaining 1499 revised models, the base coverage on each chromosome was extracted using bedtools genomecov (v2.21.0) [33]. A perl script was then used to convert genomecov’s chromosome base by base coverage to a bitmap (0 for no coverage at base position, 1 for coverage) and an array of coverage values for each exon of each of the ‘Hongyang’ 1499 revised alignments models. These were filtered to identity models incomplete coverage and models with coverage across their entire match regions. Of the 1499 revised models examined 131 possessed exons which were not supported by Red5 RNA-Seq while a further 372 revised ‘Hongyang’ models possessed RNA-Seq coverage of less than 5 reads per base. Given that we used all Red5 RNA-Seq combined in this analysis while annotators examined evidence library by library it is possible that these were not annotated due to inconsistent coverage across evidence libraries. The remaining 1069 regions represented 967 individual revised models each with RNA-Seq coverage of greater than 5 reads per base. We have provided a list of those revised models in Additional file 6 including their locations in the Red5 whole genome sequence and the average number of RNA-Seq reads aligned on a per base in each CDS.

In order to further assess our manual annotation gene set relative to the existing gene sets for ‘Hongyang’ we compared each set with 812, bidirectionally sequenced, cDNA clones generated from Hort16A cDNAs selected in the *A. chinensis* EST sequencing program [25] (Additional file 7). When these 812 cDNA sequences were aligned to the gene sets of Red5 and ‘Hongyang’ 12, 58, and 39 did not possess a match to any CDS within the Red5, original and revised ‘Hongyang’ gene sets respectively while 635, 465 and 510 cDNAs aligned to these gene sets, respectively with 60% or greater identity (Table 3a). The longest ORF for each of the 812 cDNAs was extracted and the protein set was further reduced to 550 sequences (Additional file 8) by culling any sequence not likely to encode a full length protein sequence based on comparison to NCBI RefSeq Plant (version 76). These 550 protein sequences were compared to protein predictions of both ‘Hongyang’ and Red5 models and the best match identified for each of the 550 test set proteins. Of the 550 proteins in the test set 3, 12, and 9 possessed no protein match in the Red5, original, and revised ‘Hongyang’ gene sets respectively (Table 3b) while 144, 29, and 51 proteins from the test set possessed 100% identity to a protein within the Red5, original, and revised ‘Hongyang’ gene sets respectively (Table 3c). The number of matches with identity of 95–99% in Red5 was double that for the original ‘Hongyang’ gene set (Table 3c) while it was almost 1.5× that for the revised ‘Hongyang’ gene set. Collectively these results indicate that our manual annotation has yielded a gene set considerably different from previous gene sets for ‘Hongyang’ and while the revised gene set of Yue and colleagues [18] is a vast improvement over that of Huang and colleagues [14] our analyses suggest that our manual curation has further improved the overall structure of the *A. chinensis* gene set.

**Table 3:** Alignment of DNA for 812 bidirectionally sequenced Hort1A cDNA sequences (A) and 550 Hort16A predicted protein sequences (B & C) with the Red5, original ‘Hongyang’ [14], and revised ‘Hongyang’ [18] gene model sets

	Red5 Models	Original Hongyang Models	Revised Hongyang Models
A			
Hort16A cDNA with BLAT match identity^a^ > = 60%	635	465	510
Hort16A cDNA with BLAT match identity^a^ < 60%	165	289	263
Hort16A cDNA with no match	12	58	39
	812	812	812
B			
Hort16A predicted protein with BLAT match identity^a^ > = 60%	541	462	481
Hort16A predicted protein with BLAT match identity^a^ < 60%	6	76	60
Hort16A predicted protein with no match	3	12	9
	550	550	550
C			
% Identity Range^a^			
0–59	6	76	60
60–64	2	23	7
65–69	2	21	15
70–74	5	20	16
75–79	2	36	19
80–84	13	39	28
85–89	17	54	47
90–94	17	72	67
95–99	339	168	231
100	144	29	51

^a^% identity was calculated as the number of matched bases (A) or matched amino acids (B & C) between Hort16A and the named gene set divided by the total length of the Hort16A cds sequence (A) or predicted protein sequence (B & C)

**Fig. 5:**
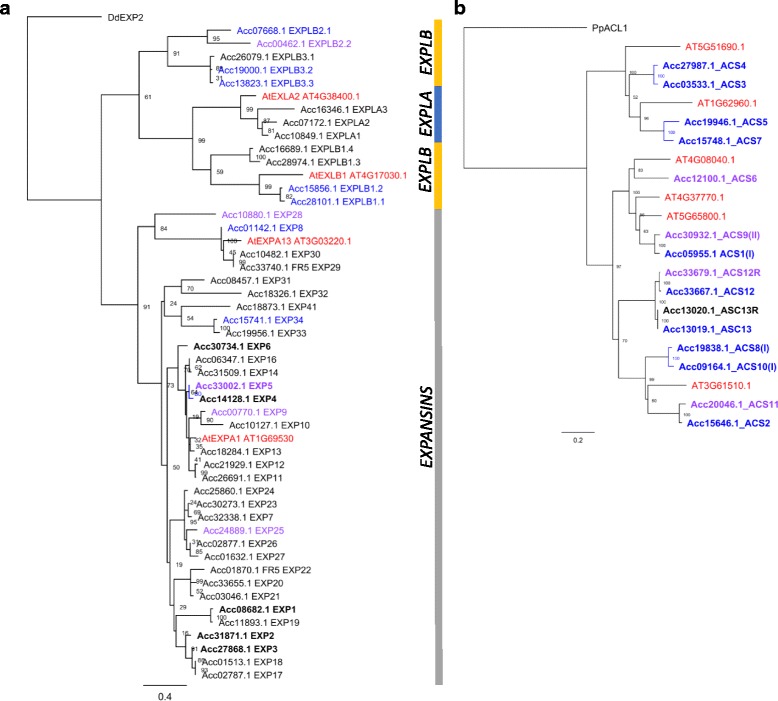
Phylogenetic alignment of alignable protein sequences from EXPANSIN and ACC SYNTHASE proteins. Proteins in red are reference *Arabidopsis* proteins*;* blue and purple are published *Actinidia chinensis* protein sequences (blue are correctly annotated published unnamed ‘Hongyang’ proteins represented by gene models, purple models are partially represented); black are models are unannotated in the 2 published ‘Hongyang’ protein sets. Each tree is rooted with a basal species. Bootstrap values of 1000 iterations are given. A. EXPANSIN proteins; vertical line represents EXPANSIN proteins (Grey) EXPANSIN LIKE B proteins (Yellow) and EXPANSIN LIKE A proteins (blue). Rooted with DdEXP2 (NCBI # gi|74,854,151) B. ACC SYNTASE Proteins. Rooted with PpACL1 (NCBI# EDQ51432.1)

Using the new manually annotated gene models, two gene families were investigated further to assess whether new annotation had missed any genes. Given the reported poor annotation of *EXPANSIN* (*EXP*)-like genes [17], the genome sequence was translated into all six translation frames and used to identify regions that had homology to *Arabidopsis* EXP, and EXPANSIN-LIKE (EXPL) protein sequences. Fifty three chromosomal regions were identified, and 41 of these had a new manually annotated gene model. Of the 12 remaining gene models, six were partial regions of homology from which no gene models could be generated. Two regions each coded for a possible full length gene even though there was no associated computer-predicted model and no RNA-Seq evidence to support them. These two genes were scored Q0 and added to the gene list, producing a possible 47 *EXP* genes. Comparison of these 47 *EXP* genes with the ‘Hongyang’ gene models showed that six were identical to the ‘Hongyang’ gene models (Fig. 5a, Additional file 9), 18 were partially supported by an original ‘Hongyang’ gene model and 15 by a revised ‘Hongyang’ gene model. 29–32 EXP models were new and interestingly the majority of these were EXP genes with the EXPL more accurately predicted. A second gene family, the *ACC SYNTHASE* (*ACS*) genes, was also investigated [34]. In total 16 translated chromosomal regions showed homology to ACS proteins. Of these 16, 14 had a manually annotated gene model associated with them (Fig. 5b, Additional file 9). For the two regions that did not have a manually assigned gene, no convincing gene models could be derived. Two genes, *ACS12* and *ACS13,* had duplicate gene models that were 100% identical to each of the models at the nucleotide level, with *ACS12* and *ACS12R* on two separate scaffolds on the unassigned chromosome (Chr30), suggesting these are possibly allelic. The other pair (*ACS13* and *ACS13R*) were found as a tandem duplication on Chr12. When the 14 *ACS* genes were compared with the original ‘Hongyang’ gene models, the 12 unique genes were all found; nine of these models were identical to the manual annotation, and three had a single exon/intron boundary difference, these numbers were unchanged in the revised ‘Hongyang’ models. The *EXP* study suggests that it is likely, even after manual annotation, that there are still unannotated genes of other gene families in the genome, especially in gene families of similar structure to the *EXP* genes i.e. smaller and computationally hard to predict. However, the new manually annotated genes do contain a more comprehensive list than the previous computationally generated gene lists.

**Fig. 6:**
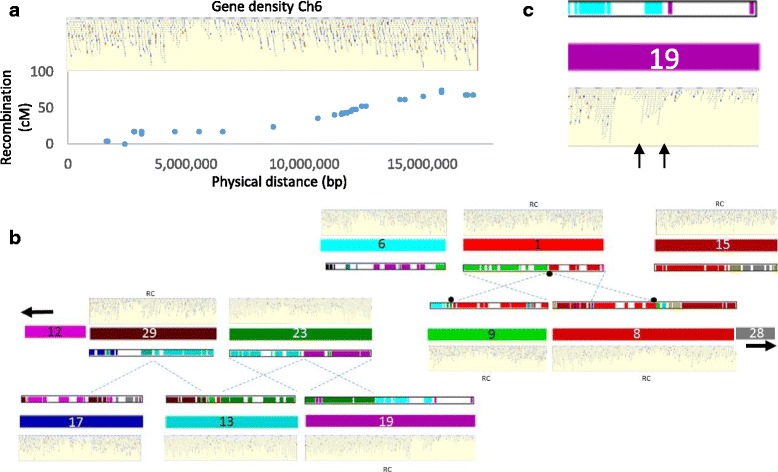
Gene density along *Actinidia chinensis* chromosomes. **a** Comparison of gene density of Chr6 (depicted by image of genes along chromosome) and physical distance measured in bp along the chromosome and genetic distance in centimorgans (cM), measured in a mapping population. **b** The relationship between gene density and regions of homology between sister chromosomes. When the gene plots were aligned to a subset of homeologous chromosome arrangements, translocation events appear to be linked to areas of lower gene density (cut point marked by small vertical arrows), RC represents gene alignment images that have been reversed to aid alignments. **c**) An expanded view of Chr19 which demonstrates two regions of low homology to other kiwifruit chromosomes, and low gene density, surrounding a region of high homology and higher gene density marked by arrows

At a chromosomal level, the gene density along the chromosomes varied, with less well populated regions associated with lower recombination rates (Fig. 6a). These lower density regions have been previously linked to centromeric regions [35, 36], and are also often associated with the translocation cut sites, consistent with the Robertsonian translocation. Especially clear are translocations of Chr1 to Chr9 and Chr8, where estimations of centromere locations can be made based on gene density (Fig. 6b). In other chromosomes such as Chr6 it is less clear, suggesting that within the same chromosome there are a number of translocation events that have occurred. Fig. 6c demonstrates that these other non-Robertsonian translocations are evident in Chr19, where low gene density regions flank a region of homology to Chr6 with a high gene density (Fig. 6c). As well as a WGD and subsequent translocations there are a considerable number of localised duplicated regions. Indeed, 1572 sites in the genome contained tandemly duplicated genes, representing 9.43% of annotated genes.

## Discussion

In this project we have developed a whole genome sequence of a second genotype of *A. chinensis* genome which, in terms of assignment of scaffolds to pseudochromosomes, assigns all but ~ 1% of assembled scaffolds to a linkage group. We also have a high quality dataset of annotated gene models using manual annotation. A more coherent genome will facilitate gene identification studies in segregating populations, and allow more accurate identification of QTLs to genomic regions and ultimately polymorphisms associated with the QTLs. Our genome incorporates ~ 73% of the estimated genome size and to improve upon that further will likely require use of long read sequence technologies. The improved gene models will greatly enhance our molecular understanding of kiwifruit and plants in general, contributing a high-quality plant gene set for the plant community for global comparisons and underpinning molecular biology in this species.

During revision of the original ‘Hongyang’ annotation, Yue and colleagues [18] adopted the identifier format “AchXXgXXXXXX” where ‘Ach’ is the abbreviation of kiwifruit species name of *A. chinensis* in three characters. The two digits of a number following ‘Ach’ denoted the chromosome, and the next letter ‘g’ identified the putative gene. This naming schema follows that for Arabidopsis. During our analysis we identified 21,479 genes having a best reciprocal BLAST match between the DNA coding regions of our Red5 model and those described by Yue and colleagues [18]. However, for 5934 of these best reciprocal gene matches there was conflict in chromosome assignment, suggesting that this was not the best method to use for genomes that are still improving. To this end we chose not to give a chromosome location as part of a gene name. This also lays the ground for the possibility for pan genome annotation within *Actinidia* species, it will accommodate further genome improvements, the issue of polyploidy, and possibility of chromosomal rearrangements that may have occurred in other *Actinidia* species. Finally given the heterozygosity of *Actinidia* species, and no current reference cultivar, we feel this naming methodology is currently the best option.

The available gene predictions from ‘Hongyang’ included only the coding regions of loci. Our visual annotation of the ends of the genes with RNA-Seq data enabled the identification of the complete transcriptional cassette in many instances. The importance of UTRs in gene regulation is becoming more and more apparent; for example, -uORFs have recently been shown to control vitamin C production in kiwifruit [37] and there is a link between introns in the 5´ UTR and transcription abundance [38]. In the manually annotated models of Red5 we observed that many of the transcripts had introns in the UTR and often these appeared to be alternatively spliced. Because of the large numbers of potential alternate UTR splice sites, as well as the ambiguity as to whether these are allelic differences, the UTR splice variants have not been included in the manually annotated gene set. These should be investigated further by other researchers when analysing individual genes.

We observed significant discrepancies between both the computer-predicted gene sets for *A. chinensis* ‘Hongyang’ and the manually annotated models for Red5. The number of changes was considerably greater than for the recent re-annotated gene models in the *Arabidopsis* gene set [19] and this degree of difference was not expected given how closely related ‘Hongyang’ and Red5 are at the genome sequence level. While *Arabidopsis* is probably the best plant gene set in terms of quality of models, the recent improved annotation process still corrected 10% of the gene models [19]. The two examples of gene families given in this paper (Fig. 5) are the extreme examples of computer prediction, the ACS genes that were accurately predicted and the EXP genes that were very poorly predicted. This annotation methodology needs to be optimised by each gene family showing a complexity required in building automated annotation pipelines. An issue noted during our study was that the increased speed of annotation conferred by having a large number of annotators was balanced by inconsistent interpretation of gene structure by individual annotators, particularly for models that were harder to interpret. The scale of these inconsistencies meant that it was necessary for a small number of ‘expert’ annotators to check each gene model (Additional file 10), adding a considerable time to the project but greatly increasing the quality of the output. The manual annotation inconsistencies were almost always around the harder to annotate (Q1) gene models, with Q2 gene models usually not needing to be adjusted. This variation among manual annotators could be addressed by more clearly setting out expectations of how to deal with conflicting data during pre-annotation training.

Our development of gene models was weighted heavily towards the use of RNA-Seq evidence, together with indications from the computational gene models. However, the RNA-Seq did not always give a clear picture of gene structure. Very often there were reads that mapped to intronic regions; sometimes these reads were specific to a single intron (Fig. 4a), and sometimes they were distributed across the whole gene (Fig. 4b). Sometimes these anomalies could be resolved using transcription data from different RNA-Seq libraries (Fig. 4c). The reads were assumed to either be part of intron read-through, or antisense transcription associated with those genes [39]. The piecing together of RNA-Seq reads in intergenic regions with no apparent open reading frame associated with them is often ambiguous. While these may be associated with non-coding RNA and they are currently left unannotated (Fig. 4d).

WGD has been proposed as promoting diversification of gene function [40]. Within the *A. chinensis* genome there is also evidence of global duplication, as well as local gene duplications. The global changes caused by Robertsonian chromosomal translocations noted in our genome assembly were originally identified in animals and often lead to birth defects. In a duplicated plant genome any such effects of these changes must be buffered and indeed have been previously reported in polyploid plants such as wheat [41], *Brassica oleracea* [42] and strawberry [43]. The duplication has caused previously identified single copy genes [44] (Table 4) to exist as two or more copies in the Red5 genome, with the exception of the three which appeared only to have a single copy. Many of the duplicates of the single copy genes were found on the respective homeologous chromosomes, as shown in bold in Table 4. The loss of function of a duplicated gene was sometimes observed with transcribed genes with no open reading frame (such as Chr1, position 10.85 Mb has a functional homologue on Chr9 (Acc09963)).

**Table 4:** Analysis of single copy genes in the *Actinidia chinensis* genome

*Arabidopsis*		Kiwifruit					
		Best hit 1	Chr	Best hit 2	Chr	Best hit 3	Chr
*AGT1*	AT2G13360	Acc30351.1	**26**	Acc32422.1	**28**		
*MAG1*	At3g47810	Acc29850.1	26	Acc04108.1	**3**	Acc02358.1	**2**
*DIENELACTONE HYDROLASE-* like	At2g32520	Acc13638.1	12				
*ATPQ*	At3g52300	Acc25925.1	23	Acc30188.1	**26**	Acc32243.1	**28**
*RIBOSOMAL PROTEIN S8e*	At5g06360	Acc12882.1	**11**	Acc17591.1	**16**		
*RRM*	At5g04600	Acc04134.1	**3**	Acc27568.1	**24**		
*MGP1*	At2g21870	Acc05500.1	5	Acc10590.1	9		
*EIF3K*	At4g33250	Acc12920.1	**12**	Acc23419.1	**20**		
*Fb15*	At4g30010	Acc12893.1	**11**	Acc17577.1	**16**	Acc27046.1	23
*CCP2*	At1g77710	Acc05095.1	**4**	Acc24370.1	**21**		
*Glycine rich protein*	At4g08230	Acc05092.1	4				
*Cytochrome c oxidase*	At4g37830	Acc05943.1	**5**	Acc30914.1	**27**		
*Unknown*	At5g47570	Acc16748.1	15	Acc15793.1	**14**	Acc19901.1	**18**
*PFD5*	At5g23290	Acc28316.1	**25**	Acc31379.1	**27**		
*Unknown*	At1g27530	Acc04831.1	**4**	Acc23863.1	**21**		
*RIDA*	At3g20390	Acc21627	19				
*Unknown*	At5g63135	Acc22027.1	19	Acc26769.1	**23**		

Best BLAST hits to the manually annotated gene models. Chromosome numbers in bold indicates the predicted homeologous chromosomes

## Conclusions

Our study provides a second genome with a high quality gene set to the kiwifruit research community, and we are confident that the 27,783 genes with a Q2 score are good quality gene models representing transcribed genes. When these are used for comparative purposes in analyses of other plant genomes, either through computational prediction or through addition of tracks for manual assignment, our models will provide users with greater confidence in their newly developed gene models. Our work highlights that a labour-intensive human intervention is still the most accurate way of predicting genes, and identifies improvements that need to be made in computational predictions of coding sequences and intron/exon boundaries.

## Methods

### Plant material

Two F_1_ diploid *A. chinensis* Planch. var. *chinensis* from an open pollinated red-fleshed fruiting mother were screened with 8 microsatellites (Additional file 11) to ascertain that they were true siblings. These were crossed and F_2_ offspring, a female with red fruit (Red Female 2) and male were selected for further crossing. Forty F_3_ progeny were sown, with each having an inbreeding coefficient of 0.375 (Fig. 1). A red fruiting female (Red5) was chosen for genome sequencing. For gene expression analysis (RNA-Seq), different tissues were harvested from mature Red5 plants to encapsulate a diversity of expression (Table 3).

### DNA isolation and sequencing

Nuclear DNA was isolated from leaf tissue of Red5 using nuclei enrichment and DNA extraction as described by Naim and colleagues [45]. DNA was sheared to an insert size of either ~ 160 bp or ~ 240 bp and prepared for 100 base paired-end sequencing along with 100 base long-insert mate-paired-end (LIMP) libraries with average insert sizes of 4, 9 and 13 Kb and sequenced on Illumina HiSeq2000™ (Illumina Inc. San Diego, CA, USA) at the Australian Genome Research Facility (AGRF - Brisbane), according to the manufacturer’s instructions. A 4 Kb insert library was also prepared for paired-end sequencing by Life Science (Roche) 454 GS-FLX. Cyclically corrected sequences from a small number (6) of PacBIO SMRT cells (45 Mb per cell) were also included during gap closing.

### RNA isolation and sequencing

RNA was extracted using the method described in Chang and colleagues [46]. RNA samples were quantified and sample purity was verified by using a Nanodrop ND-1000 spectrophotometer (Thermo Fisher Scientific). RNA integrity was checked by an Agilent 2100 Bioanalyzer (Agilent Technologies, Palo Alto, CA, USA). RNA was supplied to Macrogen Inc. (Seoul, South Korea) for standard RNA-Seq preparation and sequenced using the Illumina HiSeq2000™ yielding either single or pair-end RNA-Seq reads.

### Bacterial artificial chromosome (BAC) library sequencing

A total of 11,520 clones from a BAC library made from nuclear DNA of the F1 mother (Red Female 1) were selected for sequencing. The re-arrayed BAC clones were grown in 96-well plates containing 1.2 mL LB liquid medium with 12.5 μg/mL tetracycline at 37 °C in an orbital shaker at 180 rpm for 16 h. The bacterial cells were harvested at 3000 rpm at room temperature for 30 min in a benchtop centrifuge. The BAC DNA was extracted using a plate based alkaline lysis method [47] and dissolved in 150 μL 28 mM Tris-HCl pH 8, 1 mM EDTA, 0.6 mM cresol red (to provide a visual aid for robotic transfers). Each BAC was individually barcoded using an in-house method BACRB (details of which can be supplied on request to the corresponding author). The barcoding oligonucleotides (BioSearch Technologies, Novato, CA, USA) were dissolved in TE buffer (10 mM Tris-HCl pH 7.5, 1 mM EDTA) to a final concentration of 50 pmol/μL. Approximately 15 ng of BAC plasmid (5 μL) was randomly tagged by primer extension pre-amplification PCR (PEP-PCR) in 50-μL reactions using AccuPrime™ Taq DNA polymerase High Fidelity system as per the manufacturer’s instructions (Life Technologies Corporation, Carlsbad, CA, USA) and 10 pmol of a corresponding BACRB oligonucleotide. The DNA was denatured at 94 °C for 2 min and amplified using 50 cycles of: 94 °C for 40 s, a two-step annealing strategy (30 °C for 2 min, ramp at 0.1 °C/s, 48 °C for 4 min), 68 °C 60s, followed by a final extension at 68 °C for 7 min. The randomly tagged BAC amplicons of each clone were amplified with the tailed BACRB oligonucleotide in a second touchdown (TD) amplification process. One microlitre of randomly tagged BAC amplicon was added to 19 μL of PCR mix (0.6 M trehalose, 40 mM Tris-HCl pH 8, 20 mM KCl, 20 mM (NH_4_)2SO_4_, 10 μg BSA, 0.5 mM MgSO_4_, 0.2 mM dNTP, 2 pM tailed-BACRB oligonucleotide, and 0.25 unit Platinum® Pfx DNA polymerase (Life Technologies)). The touchdown amplification was performed as follows: 94 °C for 2 min, 1 cycle; (94 °C for 30s, TD 60–50 °C for 30s, 68 °C for 1 min), 20 cycles; (94 °C for 30s, 50 °C for 30s, 68 °C for 1 min), 10 cycles; and final extension at 68 °C for 7 min. The barcoded samples from each 384-well plate were pooled, concentrated, and analysed by agarose gel electrophoresis. A barcoded TruSeq library was prepared for each plate pool (30 barcoded libraries). A super pool was prepared by combining 10 barcoded libraries of plate pools. A total of three super pools were obtained, and each one sequenced separate lanes on single end mode, at Macrogen Inc. (Seoul, South Korea). The three lanes generated 576.35 million reads, comprising 58.2 Gbp.

For Roche 454 GS-FLX sequencing, 50-mL cultures were grown and extracted as described above. The DNA pellet was dissolved in 50 μL buffer (10 mM Tris-HCl, 1 mM EDTA, pH 7.5) and sent to be sequenced by 454 GS-FLX at Macrogen Inc. (Seoul, South Korea).

### Assembly

A “PseudoSanger”-like approach [26] was used to assemble two paired-end read libraries with stepwise decreasing insert size (240 and 160 bases, respectively). The libraries yielded 169,008,438 and 170,367,691 read pairs, respectively. Prior to assembly, reads were error-corrected using the error correction tool from the ALLPATHS-lg assembler [48] yielding 159,232,897 and 167,054,602 corrected read pairs, respectively. These reads were also used to estimate genome size using both preQC (https://github.com/jts/sga/wiki/preqc) from sga [49] and jellyfish (version 1.1.10) [50]. Error-corrected reads were assembled using anytag (version 2.5.2) [26]. Anytag yielded 46,117,212 fragments with a minimum length of 81 bases, maximum length of 450 bases and N50 of 275 bases. These fragments were assembled using Newbler 2.9 (Roche 454, Bradford, Connecticut, USA) with settings “-m –large –het –cpu 32”. Also included in the Newbler assembly were 1,209,245 paired end sequences from a 4-kb insert library sequenced using 454 GS-FLX pyrosequencing. UniVec_Core (ftp://ftp.ncbi.nlm.nih.gov/pub/UniVec/UniVec_Core) and common sequencing primers were used for vector trimming during Newbler assembly, while the sequence of *Escherichia coli* DH10B (NC_010473.1) was used for screening for bacterial contamination.

Reads from Illumina LIMP libraries were trimmed to 36 bases and redundant pairs removed prior to use using a custom perl script. The resulting read pairs were then used to scaffold the Newbler assembly further using SSPACE2 [27], which was then followed by two iterations of gap closure using GapCloser (v1.12; http://soap.genomics.org.cn/about.html). Two additional Illumina paired-end read libraries, not used in the anytag/Newbler-based contig assembly, were also employed during gap closure along with the assembly input read libraries. These two additional libraries possessed average insert sizes of ~ 240 bp and ~ 300 bp with read lengths of 75 bp (trimmed to 64 bases) and 150 bp (trimmed to 109 bases) respectively. Gap closure using Illumina-derived sequence reads yielded a reduction in ambiguities (N) within the assembly from 17.3 to 3.57%. Cyclically corrected sequences extracted from 6 SMRT™ cells (PacBIO) containing 35–45 Mb per cell were compared with the assembly using BLAT [51] and the resulting alignments used to further infill 199.01 Kb of genome assembly gaps. This assembly process yielded 3387 scaffolds containing 554 Mb with a minimum size of 1997 bases, a maximum size of 4,436,233 bases, a mean size of 142,646 bases, and N50 of 623,820 bases, an N90 of 140,742 and 3.54% N.

Assembled scaffolds were joined using 100 base read data from the sequencing of 11,520 BAC clones. Unique BAC clone reads were extracted and mapped to the assembly scaffolds using megablast (-W 70) [52]. A custom perl script was used to filter out reads mapping at less than 100% of their length and then to merge scaffolds determined to be co-linear. The genetic map of Scaglione and colleagues [15] was used to guide assignment of the vast majority of scaffolds to linkage groups. A few remaining unassigned contigs were assigned to linkage groups using genetic markers from two other sources (Fraser and colleagues [28] and Additional file 12). To enable its use with Red5, markers were first converted to fasta sequences. For Single Nucleotide Polymorphisms (SNP) markers a sequence consisting of the SNP plus 500 upstream and downstream flanking bases was extracted from scaffolds of ‘Hongyang’ [14], grandparent assembly Red Female 1(R Crowhurst, unpublished), an unrelated yellow fleshed *A. chinensis* assembly CK15_02 (R Crowhurst, unpublished) or the Red5 assembly herein, as appropriate. For each SNP the extracted fasta was named so as to encode the scaffold of origin, the location of the SNP in the scaffold of origin, the sequence region extracted from the scaffold of origin, the linkage group and centimorgan position from the genetic map and the map of origin. The names of the fasta sequences as described are provided in Additional file 12. For EST-based markers [25] the sequence of the EST was obtained from NCBI GenBank. The FASTA sequences for markers were aligned to assembly scaffolds for Red5 using megablast (-W 50) and filtered to remove alignments of less than 98% of overall length and identity before being used for assignment of scaffolds to linkage groups. An ‘all by all’ megablast comparison of scaffolds of ‘Hongyang’ [14] and Red5 was used to assign further Red5 scaffolds to linkage groups using BLAST walking from already assigned Red5 scaffolds. Red5 scaffolds identified as chimeric based on marker evidence (assigned to more than one linkage group or location within a linkage group) were manually inspected, split at identified break points and the component parts re-assigned as supported by evidence (Additional file 12).

To assess the level of DNA sequencing incorporation into the final assembly, the DNA sequencing libraries used as inputs to the anytag software were aligned to the chromosome assemblies using bowtie2 [53] using command line options: --end-to-end --very-fast -I 50 -X 500 --fr --threads 8. To enable comparison with ‘Hongyang’, mapping was repeated using the ‘Hongyang’ chromosomes [14] as the reference.

To assess the accuracy of the assembly, 22 clones from the BAC library of *A. chinensis* Red Female 1 were selected such that each contained sequence spanning two markers located on Chr25. DNA for each clone was individually barcoded and sequenced using the Life Sciences (Roche) 454 GS-FLX platform. The sequences were assembled using Newbler (version 2.9) and the assembled BAC contigs compared with the Red5 genome assembly using megablast with a word size set at 50. Results were filtered to remove regions of repetitive sequence alignment or alignments with less than 98% match to the Red5 genome sequence. Alignments were converted to GFF3 format and visualised using Geneious (versions 8.1.2) [54]. Additionally, reads from the 9Kb LIMP library were aligned to individual chromosomes for the Red5 whole genome assembly using bowtie2 [53] using the following command line options: --end-to-end --sensitive -k 5 -p 8 --rf -I 1 -X 100000. For each chromosome the distance between mate pairs was visualised using hagfish_blockplot from the software ‘hagfish’ (https://github.com/mfiers/hagfish/). Individual chromosome plots were then cut and pasted to form a montage. Each plot represents the alignment across the entire length of a chromosome. Plots were produced to a standard pixel width irrespective of chromosome length. Green regions indicate mate pairs aligning to the chromosomes within the expected distance for the library. Black indicates regions without mate pair alignment. Pinkish-red indicates regions where the distance between mated paired end reads is shorter (assembly compression relative to physical genome) or greater (assembly expansion relative to physical genome).

### Mapping of Red5 RNA-Seq libraries to assembly

The RNA-Seq reads were mapped to the chromosome assemblies of Red5 using the STAR RNA-Seq aligner [55] (version STAR-STAR_2.4.2a) using the command line parameters “--chimSegmentMin 30 --runMode alignReads --alignIntronMin 21 --alignIntronMax 25000 --alignMatesGapMax 25000 --alignEndsType Local”. All RNA-Seq reads were trimmed by 13 bases at the 5´ end prior to use. Reads were additionally trimmed at their 3´ ends when quality score assessment with FastQC (http://www.bioinformatics.babraham.ac.uk/projects/fastqc/) gave quality values below 20.

### Gene annotation

Manual curation of gene models was performed as follows. The published gene models for ‘Hongyang’ [14] and *A. chinensis* ESTs within NCBI GenBank were downloaded and mapped to the assembled pseudo-chromosome sequences of Red5 using GMAP (version 2014–10-22) [31]. The sequences of the Red5 pseudo-chromosomes were repeat masked using RepeatMasker (version open-4.0.5) with options “-e ncbi -pa 30 -s -nolow -species viridiplantae -a -x -poly –gff” and with the RepBase (http://www.girinst.org/) RepeatMasker libraries (20140131). Ab initio gene model prediction was performed using Augustus-3.1 [56] employing command line options “--species = arabidopsis” and evidential hints from both RNA-Seq derived from Red5 (Table 3) and from 47,384 *A chinensis* EST sequences (downloaded from NCBI GenBank) using described protocols (http://bioinf.uni-greifswald.de/bioinf/wiki/pmwiki.php?n=Augustus.Augustus). The genome sequence, ab initio predicted models, mapped ESTs and ‘Hongyang’ gene models as well as RNA-Seq alignments were imported into WebApollo1 [21] and made available for community-based manual curation via Amazon Cloud. Where evidence suggested multiple isoforms that could clearly be defined each isoform was included. Where it was not possible to differentiate individual isoforms unambiguously a single model was submitted by community annotators. After initial annotation, all models were ported to WebApollo2 (version 2.0.2) (https://github.com/GMOD/Apollo/releases/tag/2.0.2) and each annotation was reviewed manually. This review was followed by computational analysis to identify anomalous annotations such as small (< 15 bp) introns and untranslated exons numbering more than 3. These models were rechecked and often further modified or removed. The manually annotated gene models were named using the prefix Acc (for *Actinidia chinensis* var. *chinensis*) and sequentially numbered. The cDNA, CDS, peptide and GFF3 records for each model were committed into a GitHub repository to enable tracking of changes over time. A schema for manual annotation can be found in Additional file 10.

### Estimating genome completeness using BUSCO analysis

Benchmarking Universal Single Copy Orthologs (BUSCO analysis) [30] was used to examine genome completeness. Version 2.0 of BUSCO was used with NCBI Blast+ version 2.2.30, AUGUSTUS version 3.2.2 [56] with “--species arabidopsis”, HMMER version 3.1b2 (http://hmmer.org) and the Embryophyta_odb9 dataset (http://busco.ezlab.org/datasets/embryophyta_odb9.tar.gz). BUSCO analysis was also undertaken on the ‘Hongyang’ genome [14].

### Comparisons of gene sets

To further evaluate our manually annotated gene models we compared their coding regions with the sequence of 859 clones from cDNA libraries for *A. chinensis* var. *chinensis* ‘Hort16A’, within our in-house sequence database which had been previously individually cloned and DNA extracted [25]. The cDNA clones were bi-directionally sequenced in full, using ‘Sanger’ sequencing, and then sequences with greater than 98% similarity were removed using cd-hit-est [57], yielding a comparison set of 812 cDNA sequence with minimum, maximum, and mean lengths of 247, 4506 and 1495 bases respectively and an N50 of 1653 bases (Additional file 7). Coding regions of our manual annotation data set as well as those for ‘Hongyang’ were compared with these cDNA sequences using BLAT (version 36) and GMAP as follows. The longest ORF for each of the 812 cDNAs was extracted and sequences not likely encoding a full length protein based on comparison to NCBI RefSeq Plant (version 76) were removed leaving 550 proteins. Each of the 550 protein sequences was compared to the predicted protein sequences of the 3 gene sets (Red5, original and revised ‘Hongyang’) using BLAT and the best alignments summarised using a custom perl script.

To compare whole gene sets, for each gene set pair (Red5 with original ‘Hongyang’ and Red5 with revised ‘Hongyang’) the following analyses were undertaken: (1) predicted proteins of each pair of gene sets were compared to each other using BLAT, (2) CDS sequences for each gene set pair were compared using BLAT, (3) the CDS were aligned to the genome sequence using GMAP (version 2017–06-20). A custom perl script was then used to summarise these analyses by first seeking the best protein:protein alignments, then the best CDS:CDS alignments and finally the alignment of the query to the whole genome sequence. The summarisation perl script takes into account query and target sequence lengths for protein:protein and CDS:CDS alignments as well as genome alignment co-ordinates from the GMAP alignments in order to yield the metrics for best alignments including: number of alignments assigned to bins based on percentage identity, the number showing a length variance (query equal/shorter/longer than target), number of queries encapsulated with the UTR of a target gene model, number of queries overlapping the 5 or 3 UTR of a target gene model and the number missing from results files for these analyses. Additional file 13 summarises the raw results for the best BLAT gene set match to each cDNA protein. BLAT results presented in Additional file 13 were extracted using “awk ‘{Percent=($1/$11)*100; print $10''\t''$11'' \t''$1''\t'' Percent''\t''$14''\t''$15}’” then the difference in length between summary data columns 2 and 6 added in Microsoft Excel.

### Phylogenetic analysis

Gene models were selected and aligned in Geneious (R10.0.3) (www.geneious.com) using Geneious Alignment (with free end gaps), Gap opening penalty 30, extension penalty 0 and refinement iterations 2. Alignable regions were extracted, realigned and clustered using PHYML [58] default settings. Data from 1000 bootstrap sets are presented.

## Additional files

Additional file 1: Map back rates to the Red5 genome sequence.Summary of the numbers of input reads reads that align to the RED5 genome construction (XLSX 10 kb)

Additional file 2: BAC alignment to chromosome 25.Table summarising alignments of contigs from 22 BAC clones to chromosome 25 of the Red5 assembly (XLSX 15 kb)

Additional file 3: Comparison of predicted paired end distance to genome.Heatmaps of alignment distance scores for the alignment of the read pairs from the 9Kb long-insert mate-paired-end (LIMP) library to each of the 29 chromosomes within the Red5 whole genome assembly and. Individual chromosome plots were prepared using hagfish_blockplot from the software program ‘hagfish’ (https://github.com/mfiers/hagfish/). Individual images were cropped for height (not length) then cut and pasted into a table format for easier viewing. Each image depicted the entire length of the chromosome but all images are of standard length irrespective of chromosome length. Green regions indicate mate pairs aligning to the whole genome sequence within the expected distance of the library. Black indicates regions without mate pair alignment. Pinkish-red indicates regions where the distance between mated paired end reads is shorter (assembly compression relative to physical genome) or longer (assembly expansion relative to physical genome). (PPTX 432 kb)

Additional file 4: BLASTP comparison of manually edited gene models to the revised ‘Hongyang’ gene models. List of best reciprocal BLASTp matches between the revised *Actinidia chinensis* ‘Hongyang’ genes [18]and the Red5 gene set (XLSX 436 kb)

Additional file 5: ‘Hongyang’ Gene models that align to Red5 genome.List of *Actinidia chinensis* ‘Hongyang’ genes that align to the Red5 whole genome sequence. Additional file 5A: models from original ‘Hongyang’ annotation [14]. Additional file 5B: models from revised ‘Hongyang’ annotation [18] (XLSX 19 kb)

Additional file 6: Revised ‘Hongyang’ genes omitted from the manually edited gene set. Average RNA-Seq read coverage of the 1069 KIR V2 models perfectly aligned to the Red5 genome without a protein match in the Red5 gene set. (XLSX 114 kb)

Additional file 7: Details of sequenced cDNA’s generated. Fasta formatted sequences of 812 bidirectionally sequenced expressed sequence tag clones from *A. chinensis* var. *chinensis* used in evaluating manually annotated gene models of Red5. (FASTA 1204 kb)

Additional file 8: Sequenced cDNA’s used to verify the gene models.Fasta formatted predicted protein sequences of 550 bidirectionally sequenced expressed sequence tag clones from *A. chinensis* var. *chinensis* used in evaluating manually annotated gene models of Red5. (FASTA 220 kb)

Additional file 9: Selected gene models used for phylogeny.Table of EXPANSIN genes and ACS genes identified in this study (XLSX 19 kb)

Additional file 10: The manual annotation process.Flow diagram of manual annotation process. A. Timeline showing the manual annotation process. *see materials and methods. B. Annotation followed a 5 step process. The annotator training was completed in the form of both workshops and YouTube training videos. **https://www.youtube.com/playlist?list=PLcBe8nhQVgUg1zqOsdeRuVq9QVsLfj_Y9. (PPTX 47 kb)

Additional file 11: Parental tests of the Red5 genotype. Results from F_1_ Sibling test for the first cross. (DOCX 14 kb)

Additional file 12: Markers used in this study. List of names of extracted FASTA sequences for SNP markers used to construct the genome (TXT 841 kb)

Additional file 13: Comparison of Actinidia EST’s to manually annotated gene models.Summary of the raw results for the best BLAT gene set match protein of the sequenced cDNAs and manually annotated predicted peptides. (XLSX 102 kb)

## Abbreviations

ACS: ACC Syntase
BLAST: Basic Local Alignment Search Tool
BUSCO: Benchmarking Universal Single-Copy Orthologue
CDS: Coding Sequence
EST: Expressed Sequence Tag
EXP: Expansin
WGD: Whole genome duplication
