# Supplementary figures and images for: A manually annotated Actinidia chinensis var. chinensis (kiwifruit) genome highlights the challenges associated with draft genomes and gene prediction in plants
Source: BMC Genomics. 2018 Apr 16;19:257. doi: 10.1186/s12864-018-4656-3 (PMC5902842; doi:10.1186/s12864-018-4656-3)

## Slide 1
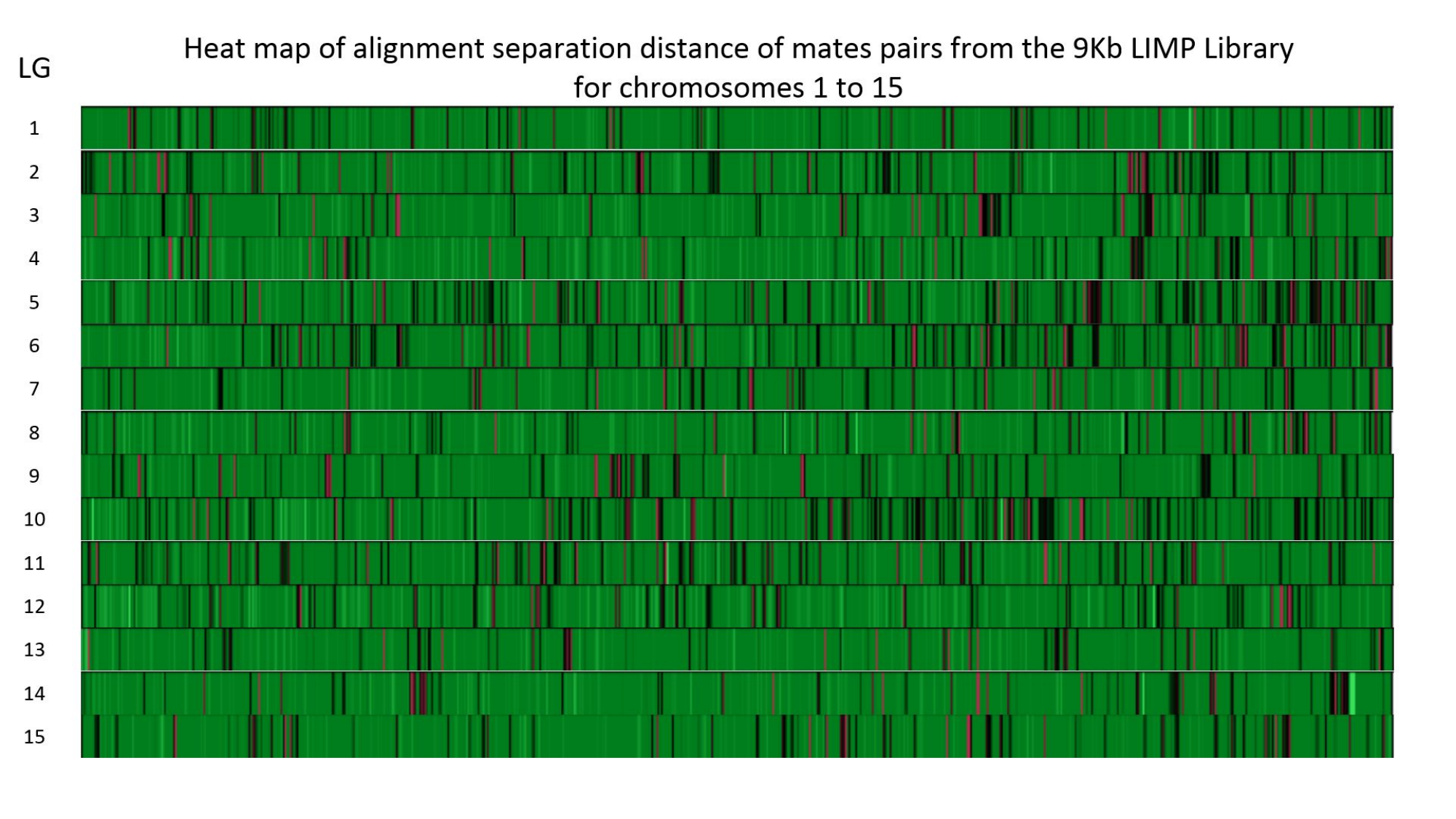

## Slide 2
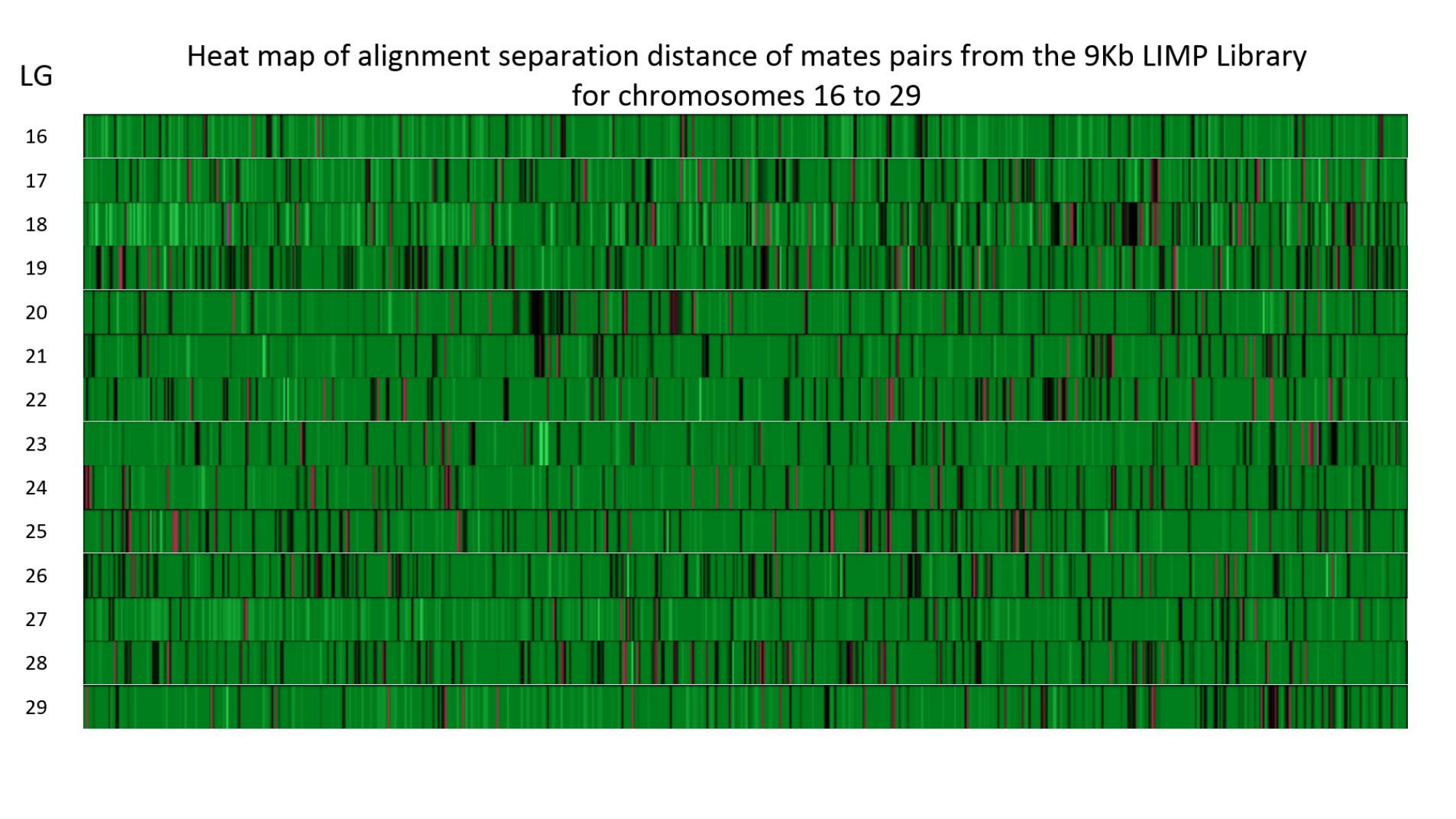

Supplement: Supplementary file 3 — Comparison of predicted paired end distance to genome.Heatmaps of alignment distance scores for the alignment of the read pairs from the 9Kb long-insert mate-paired-end (LIMP) library to each of the 29 chromosomes within the Red5 whole genome assembly and. Individual chromosome plots were prepared using hagfish_blockplot from the software program ‘hagfish’ (https://github.com/mfiers/hagfish/). Individual images were cropped for height (not length) then cut and pasted into a table format for easier viewing. Each image depicted the entire length of the chromosome but all images are of standard length irrespective of chromosome length. Green regions indicate mate pairs aligning to the whole genome sequence within the expected distance of the library. Black indicates regions without mate pair alignment. Pinkish-red indicates regions where the distance between mated paired end reads is shorter (assembly compression relative to physical genome) or longer (assembly expansion relative to physical genome). (PPTX 432 kb) [file 12864_2018_4656_MOESM3_ESM.pptx]
