# Supplementary material for: A manually annotated Actinidia chinensis var. chinensis (kiwifruit) genome highlights the challenges associated with draft genomes and gene prediction in plants
Source: BMC Genomics. 2018 Apr 16;19:257. doi: 10.1186/s12864-018-4656-3 (PMC5902842; doi:10.1186/s12864-018-4656-3)
Supplement: Supplementary file 10 — The manual annotation process.Flow diagram of manual annotation process. A. Timeline showing the manual annotation process. *see materials and methods. B. Annotation followed a 5 step process. The annotator training was completed in the form of both workshops and YouTube training videos. **https://www.youtube.com/playlist?list=PLcBe8nhQVgUg1zqOsdeRuVq9QVsLfj_Y9. (PPTX 47 kb) [file 12864_2018_4656_MOESM10_ESM.pptx]

## Slide 1
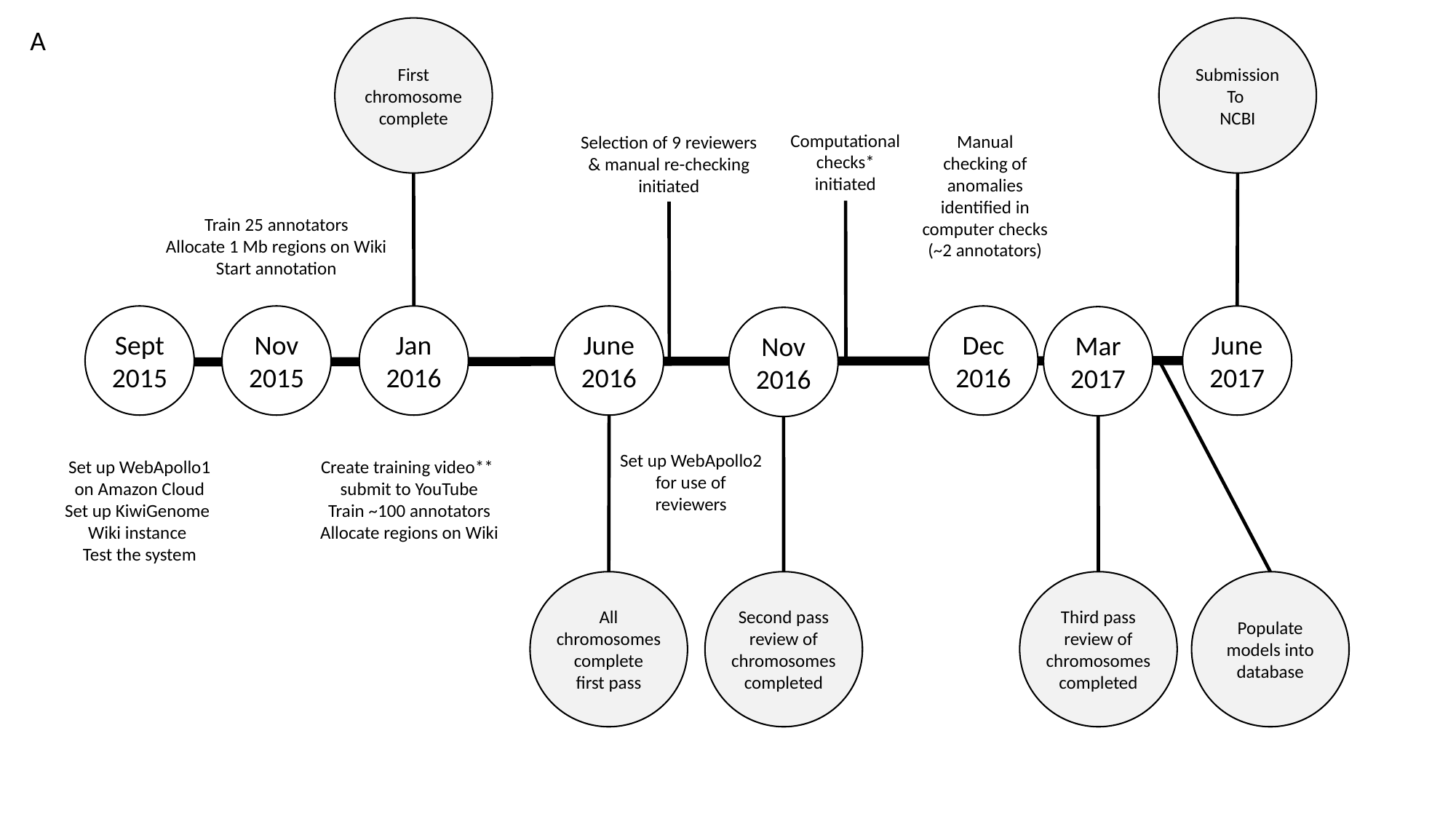

A
First chromosome
complete
Submission
To
NCBI
Computational
checks*
initiated
Manual
checking of
anomalies identified in computer checks
(~2 annotators)
Selection of 9 reviewers
& manual re-checking
initiated
Train 25 annotators
Allocate 1 Mb regions on Wiki
Start annotation
Sept 2015
Nov 2015
Jan 2016
June 2016
June2017
Dec 2016
Mar 2017
Nov2016
Set up WebApollo2
for use of
reviewers
Set up WebApollo1
on Amazon Cloud
Set up KiwiGenome
Wiki instance
Test the system
Create training video**
submit to YouTube
Train ~100 annotators
Allocate regions on Wiki
All chromosomes
complete
first pass
Second pass
review of
chromosomes
completed
Third pass
review of
chromosomes
completed
Populate models into database

## Slide 2
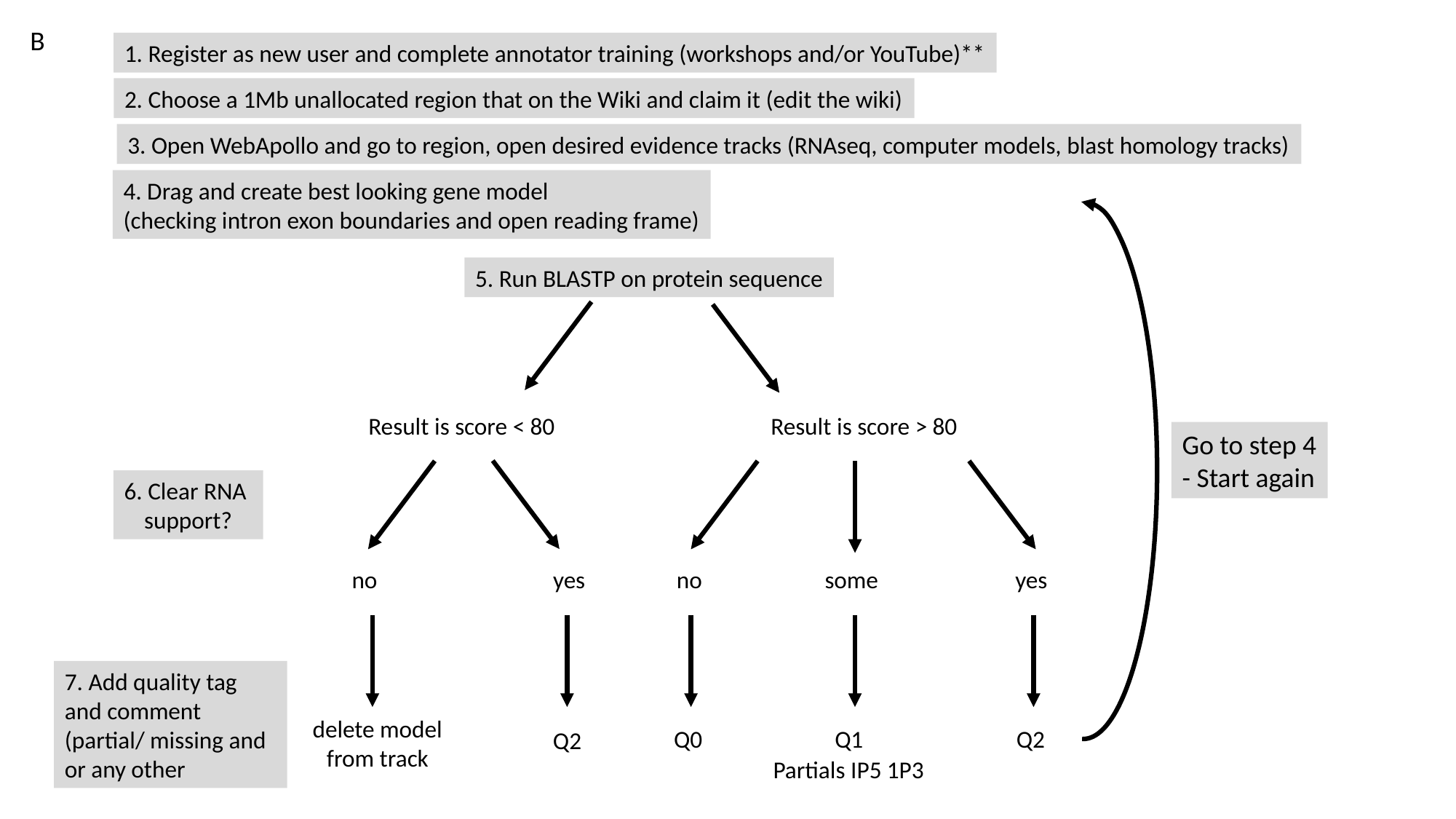

B
1. Register as new user and complete annotator training (workshops and/or YouTube)**
2. Choose a 1Mb unallocated region that on the Wiki and claim it (edit the wiki)
3. Open WebApollo and go to region, open desired evidence tracks (RNAseq, computer models, blast homology tracks)
4. Drag and create best looking gene model
(checking intron exon boundaries and open reading frame)
5. Run BLASTP on protein sequence
Result is score > 80
Result is score < 80
Go to step 4
- Start again
6. Clear RNA
support?
no
yes
no
some
yes
7. Add quality tag
and comment (partial/ missing and or any other
delete model
from track
Q0
 Q1
Q2
Q2
Partials IP5 1P3
