# Supplementary material for: A manually annotated Actinidia chinensis var. chinensis (kiwifruit) genome highlights the challenges associated with draft genomes and gene prediction in plants
Source: BMC Genomics. 2018 Apr 16;19:257. doi: 10.1186/s12864-018-4656-3 (PMC5902842; doi:10.1186/s12864-018-4656-3)
Supplement: Supplementary file 11 — Parental tests of the Red5 genotype. Results from F1 Sibling test for the first cross. (DOCX 14 kb) [file 12864_2018_4656_MOESM11_ESM.docx]

**Supplemental data 11.** Genotypes for 8 SSR markers on 31 members from the Red Ancestor family showing the inferred genotypes for the two parents, the expected and observed numbers of progeny in each of the two, three or four progeny classes, and the genotypes of Red Female 1 (RF1) and Male 1 (M1) demonstrating that they are full-sibs.

|  |  | SSR1 |  |  | SSR2 |  |  | SSR3 |  |  | SSR4 |  |
| --- | --- | --- | --- | --- | --- | --- | --- | --- | --- | --- | --- | --- |
| mother | a(331)b(343) | | | a(139)a(139) | | | a(104)b(125) | | | a(155)b(215) | | |
| father | a(331)b(343) | | | a(139)b(152) | | | c(113)d(121) | | | c(206)c(206) | | |
|  | exp |  | obs | exp |  | obs | exp |  | obs | exp |  | obs |
| progeny 1 | ¼ | 331 | 8 | ½ | 139 | 18 | ¼ | 104/113 | 6 | ½ | 155/206 | 12 |
| progeny 2 | ½ | 331/343 | 12 | ½ | 139/152 | 13 | ¼ | 125/113 | 7 | ½ | 215/206 | 19 |
| progeny 3 | ¼ | 343 | 11 |  |  |  | ¼ | 104/121 | 8 |  |  |  |
| progeny 4 |  |  |  |  |  |  | ¼ | 125/121 | 10 |  |  |  |
| RF1 |  | 331 |  |  | 139/152 |  |  | 125/121 |  |  | 215/206 |  |
| M1 |  | 331/343 |  |  | 139 |  |  | 125/121 |  |  | 215/206 |  |
|  |  | SSR5 |  |  | SSR6 |  |  | SSR7 |  |  | SSR8 |  |
| mother | a(314)a(314) | | | a(258)a(258) | | | a(285)a(285) | | | a(413)a(413) | | |
| father | a(314)b(317) | | | b(271)c(290) | | | a(285)b(297) | | | b(415)c(423) | | |
|  | exp |  | obs | exp |  | obs | exp |  | obs | exp |  | obs |
| progeny 1 | ½ | 314 | 12 | ½ | 258/271 | 20 | ½ | 285 | 19 | ½ | 413/415 | 15 |
| progeny 2 | ½ | 314/317 | 19 | ½ | 258/290 | 11 | ½ | 285/297 | 12 | ½ | 413/423 | 16 |
| progeny 3 |  |  |  |  |  |  |  |  |  |  |  |  |
| progeny 4 |  |  |  |  |  |  |  |  |  |  |  |  |
| RF1 |  | 314/317 |  |  | 258/271 |  |  | 285 |  |  | 413/415 |  |
| M1 |  | 314/317 |  |  | 258/271 |  |  | 285 |  |  | 413/423 |  |
